# Insomnia Might Influence the Thickness of Choroid, Retinal Nerve Fiber and Inner Plexiform Layer

**DOI:** 10.3390/brainsci10030178

**Published:** 2020-03-19

**Authors:** Cigdem Sahbaz, Ahmet Elbay, Mine Ozcelik, Hakan Ozdemir

**Affiliations:** 1 Department of Psychiatry, Faculty of Medicine, Bezmialem Vakıf University, Istanbul 34093, Turkey; 2 Department of Ophthalmology, Bezmialem Vakıf University, Istanbul 34093, Turkey; draelbay@yahoo.com (A.E.); hozdemir72@hotmail.com (H.O.); 3 School of Medicine, Bezmialem Vakıf University, Istanbul 34093, Turkey; aysemineozcelik@gmail.com

**Keywords:** OCT, insomnia disorder, sleep, retinal nerve fiber layer, choroid, inner plexiform layer

## Abstract

Sleep may play a fundamental role in retinal regulation and the degree of retinal variables. However, no clinical study has investigated optical coherence tomography (OCT) parameters in patients with primary insomnia. All participants were evaluated with the insomnia severity index (ISI) and the Pittsburgh sleep quality index (PSQI). The retinal nerve fiber layer (RNFL), ganglion cell layer (GC), inner plexiform layer (IPL), macula and choroidal (CH) thickness were compared between 52 drug-naïve patients with primary insomnia and 45 age-gender-BMI-smoke status matched healthy controls (HC). The patients with primary insomnia differed from the HC regarding RNFL-Global (*p* = 0.024) and RNFL-Nasal inferior (*p* = 0.010); IPL-Temporal (*p* < 0.001), IPL-Nasal (*p* < 0.001); CH-Global (*p* < 0.001), CH-Temporal (*p* = 0.004), CH-Nasal (*p* < 0.001), and CH-Fovea (*p* = 0.019). ISI correlated with RNFL-Global and RNFL-Nasal inferior. The regression analysis revealed that ISI was the significant predictor for the thickness of RNFL- Nasal inferior (*p* = 0.020), RNFL-Global (*p* = 0.031), and CH-Nasal (*p* = 0.035) in patients with primary insomnia. Sleep disorders are seen commonly in patients with psychiatric, including ocular diseases. Adjusting the effect of insomnia can help to clarify the consistency in findings of OCT.

## 1. Introduction

Insomnia, persistent difficulty in initiating or maintaining sleep and corresponding daytime dysfunction, is a major public health issue and a common disorder associated with adverse long-term medical and psychiatric outcomes [1]. The prevalence of insomnia in the general population ranges between 8–40%, while the 20–30% of the general population has poor sleep [2], and the underlying pathophysiological mechanisms and causal relationships of insomnia with diseases are poorly understood [3]. The retina is a part of the central nervous system (CNS) and perceived as a “window to the brain” [4] in establishing similarities in the physiology and function of the CNS [5]. The retina also contains a circadian rhythm, and several studies have identified that many aspects of retinal physiology and functions are under the control of a retinal circadian system [6,7,8].

The release of the two primary neurotransmitters, dopamine (DA) and melatonin (MLT), provide the “day” and “night” signals in the retina, which reconfigure retinal circuits and shape the functioning of the retina according to the time of the day [6,9]. Dysfunction of the circadian rhythm within the retina affects adversely the retinal function in the processing of the light information, synaptic communication, and metabolism [8]. Taken together, sleep disturbances might have a role in retinal regulation and the grade of retinal variables.

Optic coherence tomography (OCT) provides a promising non-invasive and feasible methodological approach for investigating abnormalities in systemic conditions where possibly the degenerative changes are related to the optic nerve and retinal architecture [10]. Initial applications of OCT were limited mainly to ophthalmic diseases, but several studies found that the changes of the neural layers of the retina might predict CNS pathology such as cortical atrophy with patients in many non-ocular diseases [11,12]. Similarly, several studies on OCT tested the progression of the psychiatric disorders and found the thinning of RNFL or macula in patients with schizophrenia (SZ) [13], bipolar disorder (BP) [14], major depression (MD) [15], and anorexia nervosa [16]. However, the retinal findings were not strongly correlated with clinical symptoms in major psychiatric diseases [10]. Hence, the researchers suggested that the lack of consideration of potential confounder factors might explain the observed heterogeneity of the results of OCT parameters such as having metabolic diseases and using psychiatric medications [13].

According to our hypothesis, insomnia might be a potential confounder factor for the OCT findings in patients with major psychiatric disorders. A growing body of evidence demonstrates that sleep and circadian rhythm disruptions are associated with the pathophysiology of psychiatric [17] and neurodegenerative disorders [18,19]. Recent Genome-wide association analysis identified 57 loci associated with insomnia symptoms and asserted the evidence of shared genetic factors between insomnia and cardio-metabolic, behavioral, psychiatric, and reproductive traits [20]. Even more, studies among patients with ocular diseases such as blindness, glaucoma [21], and central serous chorioretinopathy (CSCR) [22] have reported a higher prevalence of sleep disturbances. Evidence supporting this hypothesis is coming from studies in which the obstructive sleep apnea syndrome (OSAS) recognized and treated showed that better anatomical and functional visual outcomes in patients with CSCR [23] and age-related macular degeneration [24] found after treatment. OSAS is a chronic respiratory-related sleep disorder and recognized as a risk factor for many systemic disorders, including hypertension, cardiovascular disease [23]. The respiratory component of OSAS may produce significant hypoxic tissue responses; however, it has not been investigated whether the retinal changes are caused by sleep component or respiratory component [25].

To the best of our knowledge, there is no study conducted to investigate the direct effect of sleep disorder such as primary insomnia on the retinal findings excluding the potential confounders. Therefore, we aim to explore the OCT parameters in fifty-two drug-naïve patients with primary insomnia compared with age-BMI-gender-smoking matched healthy controls. All participants were excluded from potential confounders such as relating primarily to ocular disorders, neuropsychiatric disorders, metabolic diseases, and drug use.

## 2. Materials and Methods

The sample consisted of 52 participants with primary insomnia, and 45 healthy control subjects, aged between 18 and 65 old. All participants were recruited from the psychiatry and ophthalmology outpatient clinics of a university hospital, i.e., Bezmialem Vakif University (Istanbul, Turkey). All participants were interviewed by senior psychiatrists. The primary insomnia was diagnosed based on DSM-IV (APA, 2000) criteria which consist of the predominant insomnia complaint with the difficulties on initiating and maintaining sleep with subjectively experienced daytime impairments, excluding any organic origin for at least 1-month period, additionally only the patients with primary insomnia considered eligible to take part in the study.

The healthy control subjects were recruited from a pool of the administrative staff of the hospital. Participants with mental retardation, psychotic disorders, mood disorders, obsessive compulsive disorder, substance use and dependence were excluded. Other exclusion criteria were as follows: (i) Any medical diagnoses, e.g. diabetes mellitus, hypertension, and metabolic syndrome, OSAS, (ii) neurodegenerative diseases, (iii) pathologies of the eye, anterior and posterior segment diseases including a history of ocular contusion, cataracts, glaucoma, corneal diseases, uveitis, macular degeneration, diabetic retinopathy, retinal diseases, amblyopia, neurologic disorders such as optic neuritis; also patients who had any previous ocular operation or trauma history were excluded.

The Medical Ethical Review Committee of the Bezmialem Vakif University approved the study (Date: 01.08.2017, Number 13/204) which was conducted according to the latest version of the Declaration of Helsinki. Written informed consent was obtained from all participants.

### 2.1. Procedure

**Figure 1:**
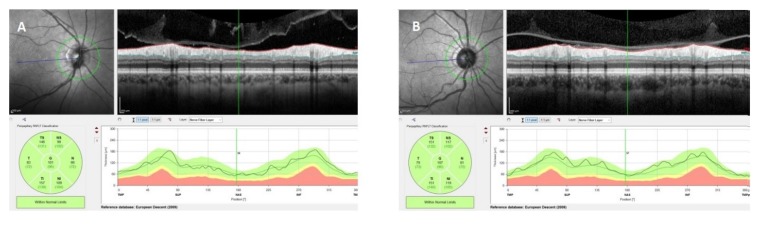
Peripapillary retinal nerve fiber layer (RNFL) thickness measurement. (**A**). A patient with primary insomnia. (**B**). A healthy individual. Top-left image: Scanning laser ophthalmoscopy image of the optic nerve. Green circle shows the corresponding OCT- circle scan shown in top-right image. Bottom-left image: Thickness map of the peripapillary RNFL thickness. While the inner 1-mm circle was defined as global RNFL peripapillary six area defined as temporal superior, temporal, temporal inferior, nasal inferior, nasal and nasal superior. Global RNFL was the mean value of these six regions.

All participants underwent a comprehensive ophthalmic examination, including corrected visual acuity measurement (with Snellen chart), slit-lamp biomicroscopy, intraocular pressure measurement and indirect ophthalmoscopy. Patients who had a spherical refractive error < −2 D or > +2 D and a visual acuity less than 1.0 were excluded from the study. All participants underwent OCT measurements including IPL, GCL, and choroidal thickness and peripapillary RNFL by the same experienced operator at the same time of the day (08:00 am–10:00 am). OCT measurements were performed without pupil dilation by using a Spectralis OCT device (software version 6.9, Heidelberg Engineering, Heidelberg, Germany). For peripapillary retinal nerve fiber layer (RNFL) thickness measurement, circular scans of 3.4 mm diameter centered on the optic disc were acquired. The images were automatically segmented into seven segments using the Heidelberg Eye Explorer software (version 1.9.10.0; Heidelberg Engineering) (Figure 1).

**Figure 2:**
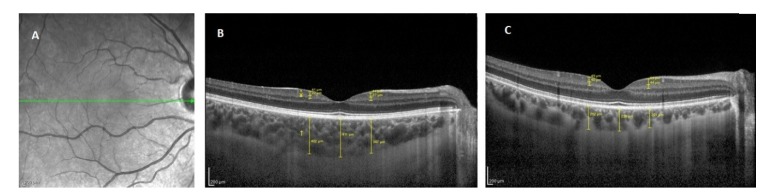
Ganglion cell layer, inner plexiform layer and choroidal thickness measurements. (**A**). Scanning laser ophthalmoscopy image of the macula. Green line is showing the cross-section of a optic coherence tomography (OCT) B-scan like in B and C. (**B**). OCT B-scan of a patient with primary insomnia. (**C**). OCT B-scan of a healthy control. *: Ganglion cell layer, ¥: Inner plexiform layer, †: Choroid.

To evaluate the GCL and IPL thickness measurement the horizontal scan crossing through the fovea was taken as a screening line. GHT and IPT thicknesses were measured from nasal and temporal points at a distance of 1000 µm to the fovea. Measurements were made by using the magnification option in the device software to enlarge four-fold the original image. As the GCL, the relative hyperreflective area between the bottom edge of the RNFL, which is the most hyperreflective band on the surface of the retina, and the upper edge of the hyporeflective area, the IPT, was accepted. The area between the bottom edge of GHT and the upper edge of the inner nuclear layer, which is a relatively hyperreflective area, was defined as IPT. To evaluate the choroidal thickness, OCT scans were acquired through the fovea with the horizontal 30-line-scan enhanced depth imaging mode of the device. The images were averaged over 100 scans using an automatic real-time imaging value of 100 and active eye-tracking features. Choroidal thickness measurements were made manually at the central fovea and at 1000 μm nasal and temporal points to the fovea. The mean value of these three measures was accepted as the choroidal thickness. The manual callipers and 2× magnification option provided with the device software were used. The distance between the outer part of the hyperreflective line corresponding to RPE-BM and the hyporeflective line corresponding to the choroid-scleral junction was evaluated as choroidal thickness (Figure 2).

### 2.2. Clinical Measurments

Insomnia severity index (ISI) quick inventory: The ISI is a seven-item questionnaire measuring insomnia symptoms and their impact on daytime functioning [26]. Scores range from 0–7 as no insomnia, 8–14 as sub-threshold, 15–21 as moderate, and 22–28 as severe insomnia.

Pittsburgh sleep quality index (PSQI): The Pittsburgh sleep quality index (PSQI) [27] was developed to evaluate the subjective sleep quality over the past month. PSQI composed of a total of twenty-four items, although the quality of sleep is calculated only by nineteen items that are self-rated. The seven-component scores range from 0 to 21 in total; higher scores indicate worse sleep quality.

### 2.3. Statistical Analysis

Demographic and clinical data of the participants were analyzed by descriptive statistics. Student *t*-tests were conducted to compare groups on continuous variables and chi-square analyses were used to compare groups on categorical variables. Data were checked whether they were normally distributed, and all relevant statistical analysis were conducted accordingly. For group comparisons on the OCT measurements, post-hoc corrections for multiple comparisons were not done due to the exploratory nature of the study. Spearman correlations were performed to analyze the relationship between the clinical variables of sleep and OCT parameters in the patient with primary insomnia and HC separately. Multiple stepwise regression analyses were conducted for each dependent variables and selection continued until all of the variables were either included or excluded. The predictor variables were stated each of the OCT measurements (as depended variables). All analyses were performed using IBM SPSS for Mac, Version 22.0 and statistical significance was set at a *p*-value of 0.05.

## 3. Results

### 3.1. Sample Characteristics

The demographic and clinical variables of the participants is shown in Table 1. The groups did not differ from each other in terms of age, gender, and BMI. According to the specified cut off scores of the ISI, 30 (26.1%) of the participants were suffering from a mild, 31 (27.0%) from a moderate, and 18 (15.7%) from a severe insomnia (Table 1).

### 3.2. Group Comparisons according to the Optical Coherence Tomography Results

There were statistically significant differences between the patients with primary insomnia and the healthy control in terms of the RNFL-G (*z* = −2.260, *p* = 0.024) and RNFL-NI (*z* = −2.591, *p* = 0.010) measurement. All areas of RNFL measurement are presented in Table 2.

**Figure 3:**
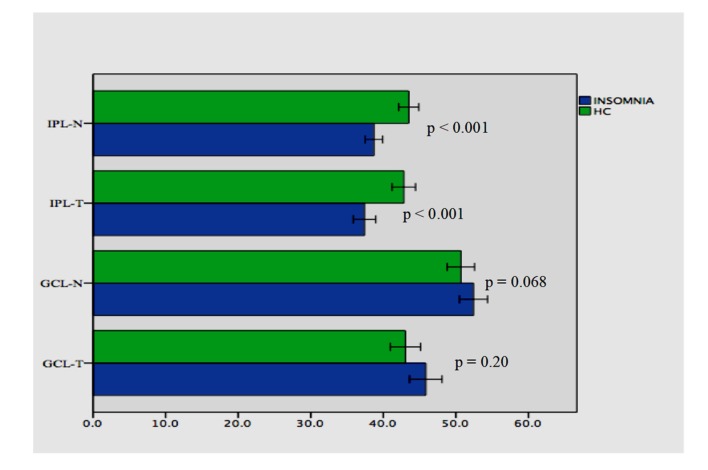
Comparisons of GCL and IPL between patients with primary insomnia and healthy controls. Abbreviations: IPL-N, inner plexiform layer- nasal; IPL-T, inner plexiform layer-temporal; GCL-N, ganglion cell layer-nasal; GCL-T, ganglion cell layer-temporal; HC, healthy controls.

There were statistically significant differences between the patients with primary insomnia and the healthy control in terms of the IPL-N (38.69 ± 4.36 vs. 43.51 ± 4.58; *t* = −5.282, *p* < 0.001) and IPL-T (37.40 ± 5.54 vs. 42.82 ± 5.36; *t* = −4.880, *p* < 0.001) measurement. There were no statistically significant differences between the patients with primary insomnia and the healthy control in terms of the GCL-N (52.42 ± 6.92 vs. 50.68 ± 6.29; *t* = −5.282, *p* = 0.20) and GCL-T (45.84 ± 8.02 vs. 43.04 ± 6.92; *t* = −4.880, *p* = 0.068). Comparisons of GCL and IPL measurements are presented in Figure 3.

**Figure 4:**
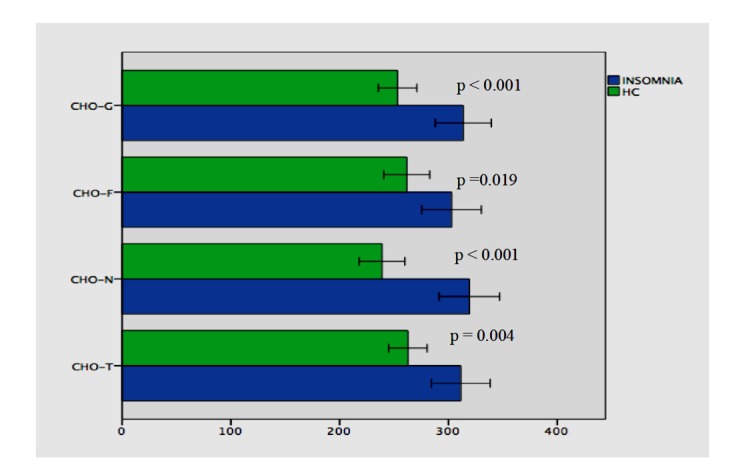
Comparisons of CHO-N (319.28 ± 100.01 vs. 238.95 ± 69.92); CHO-T (311.38 ± 97.34 vs. 262.75 ± 58.91); CHO-F (302.90 ± 98.48 vs. 261.66 ± 70.32); CHO-G (313.65 ± 92.46 vs. 253.21 ± 58.87) between patients with primary insomnia and healthy controls.

The patients with primary insomnia and healthy controls differed statistically significant from each other in terms of all choroidal measurements (Figure 4).

### 3.3. Correlation of the Clinical Variables with the Optical Coherence Tomography Measurements

Spearman correlation analyses were conducted between the clinical variables (ISI, PSQI, MEQ, BMI, duration of the insomnia) and the measurements of OCT in the patients with primary insomnia. The results revealed that the ISI score was correlated with RNFL-NI (Rho= −0.328, *p* = 0.020) and RNFL-G (Rho= −0.306, *p* = 0.031). The duration of the insomnia and BMI were not found correlated with any parameter. No significant correlation was found in the HC.

### 3.4. Stepwise Linear Regression Results

To examine the unique associations between the clinical variables, i.e., age, sex, BMI, PSQI, ISI, and OCT variables a stepwise linear regression analysis using the backward method was performed. The dependent variables were each of the OCT measurements. No variables were entered into the equation with the thicknesses of RNFL-TS, RNFL-T, RNFL-N, RNFL-NS, Macula, IPL-T, and GCL-T in patients with primary insomnia. Table 3 reports summary of the predictors of the stepwise linear regression analyses for the thickness of RNFL-G, RNFL-NI, RNFL-TI, GCL-N, IPL-N, CHO-G, CHO-N, and CHO-T. (Table 3).

## 4. Discussion

The major findings of this study were that patients with primary insomnia had the thinning of RNFL-G, RNFL-NI, and IPL-T, IPL-N thicknesses compared to healthy controls; the thinning of RNFL- G and RNFL-NI were correlated with the severity of insomnia. Second, our results showed the significant thickening of CHO-G, CHO-T, CHO-N, and CHO-F in patients with primary insomnia compared to healthy controls; and regression analyses indicated the ISI score as a predictor for the thickness of RNFL-NI and CHO-N in patients with primary insomnia.

Regarding our results, the global retinal nerve fiber layer thinning in patients with insomnia might be an effect of neurodegeneration or neurochemical dysregulation. Insomnia is associated with reduced brain activation, blood flow, or glucose metabolism. There is a growing body of evidence that sleep disruption may also accelerate the progression of pathology of neurodegenerative diseases via defective mitochondrial dynamics and axonal transport [28]. The RNFL is found more sensitive to vascular changes associated with gliosis and inflammation than other layers of the retina, which would influence OCT measurements [29]. Also, the RNFL is first-order neurons, with the unmyelinated ganglion cell axons that project to the lateral geniculate nucleus of the thalamus, it provides sensory input to the visual cortex, and the thinning of the retinal nerve fiber layer following by lesions of the thalamic-visual pathway in humans is hypothesized as retrograde trans-synaptic axonal degeneration (RTSD) [30,31] and identified by optical coherence tomography [31]. The thalamus is also a major region involved in the pathophysiology of insomnia, sleep-wake rhythms and hyperarousal [32]. Previous studies have reported functional and structural abnormalities in the thalamus in patients with insomnia [33,34]. The disrupted white-matter integrity of thalamus [35], the reduced bilateral thalamic grey-matter volume after sleep deprivation [33], atrophic changes [36], and the structural and metabolic alterations in the thalamus have been replicated in neuroimaging studies [35] in patients with insomnia. Therefore, RTSD may lead to the thinning of RNFL due to the effect of the thalamocortical dysfunction by insomnia [37].

We found also the nasal sector (nasal inferior) of RNFL was significantly affected in our patients. The segmentation analysis of the retinal layers might add important information about changes in the different retinal regions since the global thickness of RNFL alone may not reflect the pathophysiology of diseases [38,39]. Retinal ganglion cell axons are distributed in a specific topographic manner at the optic nerve head and the axons from the nasal and temporal segments of the retina have different anatomical microenvironments that might change the neurodegenerative pattern [40]. Many neurodegenerative disorders have been characterized by the different pattern of RGCL loss, and optic nerve degeneration [38]. For example, multiple system atrophy displays an OCT pattern more similar to Alzheimer whereas the OCT pattern of Huntington is closer to Parkinson’s disease [38]. Also, the relative sparing of the RNFL was found predominantly in the temporal quadrant in patients with PD, and in the superior and inferior RNFL quadrants in patients with AZ [35]. According to a meta-analysis, there was significant with a moderate effect size thinning of the RNFL for the global and nasal region in the patients with SZ and BD [14]. Another study found significant thinning of the nasal parafoveal RNFL in SZ compared to HC, but not in the overall or temporal region [41]. Our findings are more similar to the results of the study with the patients with SZ and BD in terms of the location of thinning of the RNFL in the global and nasal region. Although sleep disorders are seen commonly in both schizophrenia and bipolar disorder, it is difficult to interpret our data; global thinning of RNFL and the specific distribution of nasal inferior RNFL requires replication in independent samples with insomnia.

We found the thinning of IPL-T and IPL-N in patients with primary insomnia compared to HC. The retinal ganglion cell and inner nuclear layers of the retina contain functionally autonomous circadian clocks [6]. Also, dopaminergic (DA) amacrines and ganglion neurons of retina express key elements of the circadian autoregulatory gene network with the highest proportion in DA neurons (30%) and amacrine cells are inhibitory interneurons and with bipolar cells extend presynaptic dendrites to the IPL where they synapse with retinal ganglion cells [42]. Furthermore, almost all dopaminergic amacrine cells are GABAergic and are located at the boundary of inner nuclear layer (INL) and IPL and branched in S1 lamina of the IPL. Primary insomnia suggested as a hyperarousal state of the CNS and the consistent findings of proton magnetic resonance spectroscopy showed that insomnia is associated with lower GABA levels in the parieto-occipital cortex [43,44] and anterior cingulate [45] by reflecting presynaptic concentrations of GABA [46]. Therefore, our findings might reflect the effect of decreased level of GABA; and are in line with the studies that showed the dysregulation of melatonin and dopamine levels which might cause the thinning of IPL. RTSD might be also one of another cause of the atrophy of the inner retinal layers, such as the GCL and IPL. Therefore, researchers suggested that the decreases in GCL and IPL may reflect neuronal atrophy, synaptic loss and have shown that IPL measurements might be better biomarkers of symptom severity than the RNFL in patients with multiple sclerosis [47].

We found the thickening of choroidal layers (CHO-Global, Temporal, Nasal, and Foveal) statistically significant in patients with primary insomnia compared than healthy controls, and the regression analyses indicated that the age and severity of insomnia score were predictors for the thickness of CHO-Nasal in patients with primary insomnia. The choroid is likewise well established in humans to show significant diurnal alterations in thickness, and daily rhythms of light exposure are determined to perform a pivotal role in the synchronization of these circadian rhythms [48]. One of the potential mechanisms is that the light-induced increase of retinal DA [49] results in nitric oxide (NO) release and NO leads to an increase of choroidal blood flow and density [50]. Furthermore, this process is plausible in the literature and affects the choroidal bloodstream variations in feedback to shifting light conditions [50]. Previous investigators have demonstrated that the choroid has a relative peak thickness early in the morning and progressive decrease during the day to a relative nadir at 5:00 PM [50]. In our study, measurements of all individuals were performed between 8.00–10.00 in the morning. Therefore, we can assume that insomnia leads to choroidal thickness if we do not consider the measurement time as a confounding factor. Moreover, we found that the severity of insomnia is a predictor for the choroidal thickness. Insomnia is a clinical condition that affects circadian rhythm disruption and the proportion of exposure to the daylight. So the above-mentioned retinal dopamine and nitric oxide-mediated vascular responses caused by light responses may change the thickness of choroid.

There are several limitations to our study. The cross-sectional nature of the study does not allow us to explore the causal relationships between insomnia and retinal changes. We did not study with objective parameters in sleep. Therefore, future investigations should examine both subjective and objective parameters of sleep. Also, using actigraphy should be considered for having high concordance with polysomnography in healthy adults. We included the patients in the study from a single outpatient clinic, and our patients were mostly women. Therefore, a multi-center and homogeneity in gender studies are required to test better our results.

## 5. Conclusions

The inner retinal layers have been demonstrated in many disorders as a biomarker of the neurodegeneration, but the confounding factors may reveal that these findings have not been fully recognized yet. This OCT study is the first to show a significant decrease of the retinal nerve fiber and inner plexiform layers in patients with primary insomnia versus healthy controls. Our explorative data might provide a rationale for further examination of insomnia as a potential confounder factor for the OCT findings in patients with ocular and non-ocular diseases. Likewise, our study indicated that the thickness of choroid can show a statistically significant increase in patients with primary insomnia and may have implications to understand the impact of sleep upon the pathogenesis of retinal physiology.

## Figures and Tables

**Table 1:** Demographic and clinical characteristics of the studied population.

	Insomnia *N* = 52	Healthy Controls *N* = 45	χ^2^ (*df*)/t (*df*)	*p* Value
Age (mean ± SD)	43.0 ± 11.7	40.3 ± 12.2	1.125 (91.7)	0.26
Gender (female, number)	40	31	0.794 (1)	0.49
BMI	26.7 ± 5.8	27.6 ± 5.8	−0.780 (92.3)	0.43
Smoking (yes, number)	16	21	0.270 (1)	0.66
PSQI (mean ± SD)	12.3 ± 3.3	4.3 ± 2.0	14.436 (85.4)	<0.001
ISI (mean ± SD)	19.9 ± 4.2	5.7 ± 2.1	−21.577	<0.001
Duration of the insomnia (month, mean ± SD)	31.6 ± 45.2			

BMI, body-mass index; PSQI, Pittsburgh sleep quality index; ISI, insomnia severity index. Sociodemographic variables were calculated by Chi-square tests for categorical variables and *t*-test for continuous variables. The significance threshold was set at 0.05.

**Table 2:** Comparisons RNFL variables between patients with primary insomnia and healthy controls.

	Insomnia *N* = 52	Healthy Controls *N* = 45	*z*/*t*	*p* Value
RNFL-G (mean ± SD)	101.32 ± 9.12	105.48 ± 7.81	*z* = −2.260	0.024 *
RNFL-T (mean ± SD)	73.53 ± 12.35	76.53 ± 12.41	*z* = −1.299	0.194
RNFL-TS (mean ± SD)	140.94 ± 19.69	141.71 ± 19.01	*z* = −0.416	0.677
RNFL-TI (mean ± SD)	144.19 ± 26.93	139.28 ± 29.98	*z* = −0.651	0.515
RNFL-N (mean ± SD)	77.53 ± 12.88	78.35 ± 14.58	*z* = −0.916	0.360
RNFL-NS (mean ± SD)	122.28 ± 25.13	117.06 ± 25.13	*t* = 1.062	0.288
RNFL-NI (mean ± SD)	109.76 ± 21.84	118.84 ± 25.64	*z* = −2.591	0.010 *

RNFL, retinal nerve fiber layer; TS, temporal superior; TI, temporal inferior; NS, nasal superior; NI, nasal inferior. *, The significant threshold 0.05.

**Table 3:** Summary of multiple regression analysis with the OCT parameters in patients with primary insomnia (*n* = 52).

Dependent Variable	Predictor/s	B	SE	β	*t*	*p*
CHO-G	Age	−3.806	1.088	−0.451	−3.497	0.001
CHO-T	Age	−3.811	1.022	−0.474	−3.727	0.001
CHO-N	Age ISI	−4.421	0.923	−0.566	−4.731	<0.001
		−218.185	100.660	−0.260	−2.168	0.035
CHO-F	Age	−4.256	1.047	−0.506	−4.065	<0.001
RNFL-G	ISI	−25.422	11.419	−0.306	−2.226	0.031
RNFL-NI	ISI	−64.947	27.003	−0.328	−2.405	0.020
RNFL-TI	Gender	27.623	8.230	0.436	3.356	0.002
IPL-N	Gender	3.303	1.392	0.324	2.373	0.022

B = unstandardized beta coefficient; SE = standard error; β = standardized beta coefficient. CHO-N, choroid nasal; CHO-T, choroid temporal; CHO-F, choroid fovea; CHO-G, choroid global; RNFL, retinal nerve fiber layer; TI, temporal inferior; NI, nasal inferior; IPL-N, inner plexiform layer- nasal; GCL-N, ganglion cell layer-nasal; ISI, insomnia severity index.
